# Humans self-organise balance control strategies on a dynamic platform

**DOI:** 10.1038/s41598-025-09127-3

**Published:** 2025-07-08

**Authors:** Naser Taleshi, Amid Kheirandish, James M. W. Brownjohn, Sarah E. Lamb, Genevieve K. R. Williams

**Affiliations:** 1 Department of Public Health and Sport Sciences, Faculty of Health and Life Sciences, University of Exeter, St Luke’s Campus, Exeter, EX1 2LU UK; 2 Department of Engineering, Faculty of Environment, Science and Economy, University of Exeter, Streatham Campus, Exeter, EX4 4QF UK

**Keywords:** Biomedical engineering, Mechanical engineering

## Abstract

The human body continuously detects and predicts environmental disturbances and adaptively generates corrective responses to maintain standing balance. According to dynamical systems theory, these responses are self-organised, emerging naturally during human-environment interaction. Building on this, we introduce a model predictive controller (MPC) framework to simulate postural responses to environmental perturbations caused by a dynamic underfloor platform. The model uses a four-segment biomechanical system with sensory feedback and predicts the optimal response for maintaining balance, while accounting for biomechanical constraints, as the frequency of mechanical perturbation increases. The model findings, validated by the performance of nine young participants, provide evidence that indeed postural strategies emerge autonomously from the body’s dynamic interaction with the mechanical perturbation, without manual tuning. The emergent behaviour involves non-linear transitions from ankle to knee strategy, followed by transition in the relative motion between the centre of pressure and centre of mass as platform frequency increases. We demonstrate that effective models should include ankle, knee, and hip joint motion, with hip motion being less mechanically efficient in young people. The proposed framework also overcomes the limitations of traditional models which fail to capture the transitional dynamics and provides novel insights into the self-organising nature of postural responses.

## Introduction

Human balance control is a highly complex and dynamic process. The central nervous system (CNS) continuously integrates sensory inputs from mechanoreceptors in muscles, joints, tendons, ligaments, connective tissue and skin, as well as higher-order receptors in the visual and vestibular systems, to coordinate body movement and maintain upright standing posture^1,2^. Upon disturbance, the sensory system detects body motion^1^. In case of rapid disturbances, muscle spindles signal the spinal cord, which then initiates reflexive muscle contractions to counteract the disturbance. On the other hand, voluntary postural responses involve multiple CNS components processing sensory inputs in the brain, before regulating neural commands for corrective muscle activation and maintaining upright posture. This feedback-based mechanism, termed closed-loop dynamics, is crucial for precise movements like postural control^3–5^.

Balance control research has long employed in-vivo experiments and in-silico modelling. Traditional in-vivo methods involve applying discrete support surface perturbations to replicate slips, trips or pushes experienced in real-life and capture ankle and hip strategies used to maintain balance^6–11^. Studying response to such discrete perturbations helps to predict fall risk and to train fall breaking strategies. However, the non-linear nature of postural control^12,13^ suggests discrete perturbation tests might not capture all available postural strategies within human posture control system^14,15^. Dynamical systems theory views these postural strategies as self-organised emergent responses to the changes in environmental conditions^16,17^. Historically, researchers have identified this self-organising nature by studying how postural strategies transition in response to gradual changes in external ‘control parameters’^18,19^. This self-organisation is evident in non-linear transitions between postural strategies (e.g., from ankle to hip strategy) as the body adapts to perturbation frequency changes^14,20–22^. Following this line of research we, in recent experimental work, increased an underfloor platform frequency over time as the control parameter and continuously monitored transitions in postural strategies. We revealed knee strategy as a new and relevant strategy in real-life settings, and transition between ankle and knee strategies that underpins a transition between centre of pressure (COP) and centre of mass (COM) relative motion at a constant perturbation amplitude of only 2 mm and frequency of 2.11 and 2.19 Hz, respectively^15,23^.

In-silico modelling extends experimental research, deepening our understanding of the motor system. Applied to human motor control, it simulates how the CNS uses sensory information to coordinate muscles and joints for appropriate movements. Previous approaches for modelling postural control responses to platform motion have often employed the closed-loop control approach, drawing from principles in engineering control theory (ECT)^24–26^. The ECT method reflects the biological feedback mechanism that auto-corrects movements via comparing the actual movement informed by sensory inputs with a desired movement in the CNS. The precise mechanism for the nervous system’s corrective commands is still under debate. According to ECT, a controller, informed by body dynamics and sensory inputs, can generate effective commands for balance. Various models have been proposed for such a controller to simulate the CNS’s role in balance control process^6,26–28^. Common control laws in balance studies include proportional-integral-derivative (PID) and optimisation laws such as linear quadratic regulators (LQR), and model predictive controllers (MPC).

Johansson et al.^28^ were among the first to apply PID controllers for balance control, leading to their extensive use in studying sensory integration processes^25,29,30^. P, PD, and PID controllers are all types of PID-based control methods and are commonly used in postural models. A proportional (P) controller acts as a spring: increasing P gain reduces sway amplitude but increases frequency. It requires minimal tuning but cannot eliminate steady-state error. PD and PID controllers include a derivative term that provides damping by generating torque based on sway velocity ^31^, consistent with findings that velocity signals convey sufficient information about COM motion ^32,33^. PD controllers are widely used and considered biologically realistic but can be affected by feedback delays ^31,34^. PID controllers add an integral term to reduce steady-state error and suppress small residual oscillations, although minor sway is typical during quiet standing. While PID controllers have enhanced understanding of motor and sensory aspects of balance, they fail to account for key human system properties. Firstly, musculoskeletal constraints, such as joint movement and torque limits, are ignored, resulting in simulations that exceed human capabilities. Secondly, PID controllers lack adaptability; their constant gain parameters do not adjust to environmental changes. Although effective for single-inverted pendulum models, PID controllers struggle with time-varying perturbations and the recruitment of multiple degrees of freedom (DOF). To address multi-joint control, Morasso et al.^35^ proposed parallel PID controllers for individual joints, and Bilgin and Kemal^36^ introduced adaptive PID laws with dynamic gains. However, joint interdependence complicates this approach, as torque and kinematics at one joint affect others. Moreover, integrating multiple PID loops requires extensive gain tuning, adding significant complexity to the model.

Engineering optimisation (EO) is another key method for generating corrective neural commands to maintain balance^30,37–39^. EO explains many experimentally observed features of sensory integration by defining movement goals through a cost function (CF), which mathematically represents task objectives and constraints, and uses the outcome of this optimisation to derive control actions. A common approach is solving a quadratic function, such as the LQR. Kuo’s model^26^ applied an LQR to a triple-link inverted pendulum, creating joint torques for body dynamics regulation. The LQR controller minimises the CF by balancing control effort and state performance while considering biological constraints. Unlike PID controllers, LQR produces a feedback gain matrix that reflects compensatory torques across multiple joints based on their mechanical states. The controller then generates control responses by multiplying joint state errors with the gain matrix. Park^40^ analysed LQR feedback gains to assess joint contributions in balance control, showing that gains increase with perturbation magnitude, indicating ankle strategies for low magnitudes and hip strategies for higher magnitudes. While time-invariant feedback gains in LQR and PID controllers perform well for discrete perturbations, they may not capture the adaptability of biological systems in time-varying dynamic environments^15^.

Musculoskeletal modelling, grounded in anatomical and physiological principles, has advanced understanding of postural control by simulating joint kinematics, muscle activations, and body dynamics using detailed models developed in platforms such as OpenSim. For example, Jiang et al. ^41^ developed an OpenSim-based quiet stance model combining feedforward control with multisensory feedback. Shanbhag et al. ^42^ used delayed feedback and optimised parameters in a 6-DOF OpenSim model to reproduce realistic balance during quiet and perturbed standing. Koelewijn and Ijspeert ^43^ applied reflex-based control using feedback loops mimicking muscle spindles and Golgi tendon organs. While these models produce realistic physiological responses, they do not address how coordination strategies adjust under different environmental conditions. Strategy selection was investigated in OpenSim by Afschrift et al. ^44^ and Kaminishi et al. ^45^ using optimal and PD controllers. Afschrift showed that different cost functions (e.g., effort vs. instability) led to distinct strategies, while Kaminishi examined how muscle tonus variations affected strategy choice. However, these models primarily focus on short, discrete perturbations and do not address how postural strategies adapt to continuously changing environments.

Research from a dynamical systems perspective reveals that postural strategies emerge autonomously when interacting with environmental constraints^14,15,20^. These studies highlight transitions between balance strategies under time-varying platform conditions, necessitating a control system that adapts autonomously to environmental changes and maintain optimal performance. Over four decades ago, engineers developed the MPC framework that could be used to overcome the multi-joint challenges in PID laws and time-invariant gains in LQR and PID approaches. MPC reflects the predictive and adaptive capabilities of the human motor system^46^. By incorporating a dynamic internal model, MPC predicts system behaviour and generates proactive control signals to maintain balance^47^. It optimises a cost function at each step, updating predictions and control signals to adapt to time-varying perturbations. This mirrors the cerebellum’s role in predicting action outcomes based on sensory input, motor commands, and past experiences^46,48^. The cerebellum, through internal models, formulates corrective neural commands for posture and movement^49–51^, surpassing simple feedback controllers^52^. The iterative processes of prediction and correction in MPC and the cerebellum demonstrate their analogous roles in balance control^53–55^. Classical controllers such as PID and LQR lack adaptability, whereas MPC captures the predictive and adaptive nature of balance control. Aftab et al. ^47,53^ and Castano et al. ^56^ used MPC to model balance recovery from discrete perturbations. Shen et al. ^54,57^ and Inkol and McPhee ^55^ extended this to longer disturbances and captured multiple strategies. However, the strategies observed across separate simulations rather than within a continuous task. As such, the models were not evaluated under complex, evolving perturbations and do not fully reflect the adaptive, emergent nature of balance control in dynamic settings.

To address this gap, we propose a self-organising MPC framework grounded in dynamical systems theory ^18,19^, aimed at capturing transitions in postural strategies under gradually changing perturbations. Models based on self-organisation should maintain balance and exhibit coordination shifts in response to changes in a control parameter, in our case platform frequency ^58^. Building on prior MPC work, we extend it to simulate responses using a dynamic underfloor platform ^15^. This highlights the potential of adaptive frameworks to capture the non-linear, emergent nature of postural control.

## Methods: outline of the model

In a closed-loop control system, a ‘plant’ translates neural commands into movement, the ‘sensory system’ provides feedback on movement errors, and the ‘controller’ adjusts parameters to correct these errors.

### Plant: body dynamics model

**Fig. 1:**
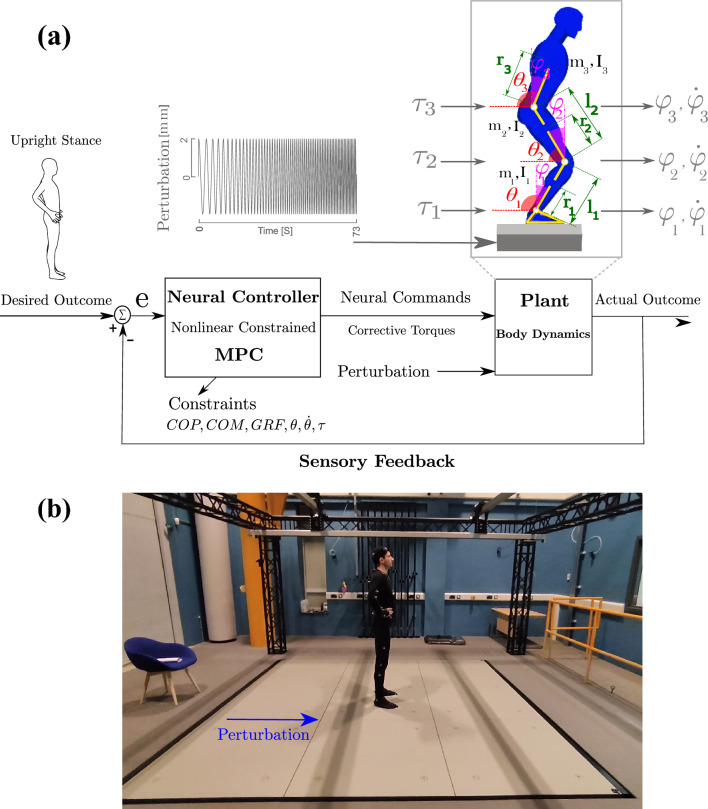
Proposed non-linear constrained MPC model to simulate balance control on a dynamic floor: (**a**) the model uses a negative feedback mechanism that senses errors and drives the desired movement based on the error. The MPC controller achieves this using optimisation techniques, allowing proactive assessment of movement trajectory and adherence to the biomechanical constraints. (**b**) Experimental Apparatus and Setup: The image depicts the moving floor embedded with force plates oscillating sinusoidally in the anterior–posterior (AP) direction. A participant stands barefoot with eyes open, and hands placed on the hips, which were not allowed to move during the standing task. Informed written consent was obtained to publish the participant’s image in an online open-access publication.

Existing models of human body dynamics, or ‘plant,’ often simplify the body as segments connected by joints. In balance control studies, these are typically reduced to single or double inverted pendulum models^59^, focusing on ankle motion in single-inverted pendulums or both ankle and hip in double-inverted pendulums. Our previous work^15^ demonstrated that a range of postural strategies (ankle, knee, hip, or combined) can emerge under different environmental conditions. Limiting knee or hip motion restricts the exploration of these strategies. To address this, we introduce a simplified 3-degree of freedom (DOF) model (ankle, knee, hip) that includes foot, shank, thigh, and HAT (head, arms, and trunk) segments in the sagittal plane (see Fig. 1). The body dynamics model is controlled by torques at the ankle, knee, and hip joints, as shown in Fig. 1. The mathematical details of the body dynamics model are provided in the Supplementary Information Appendix.

### Sensory feedback: states

The CNS integrates sensory inputs to generate corrective movements^2^. These sensory inputs, including visual, proprioceptive, and vestibular, monitor deviations from the desired body position. For example, labyrinthine receptors provide critical feedback to CNS by detecting changes in head position relative to the Earth’s vertical axis and head movement velocities. In response, the vestibulospinal reflexes stabilise the trunk via limb muscles, while the vestibulo-collic reflexes stabilise the head through neck muscles^60^. The ‘states’ of a system simplify body dynamics by focusing on key feedback elements, such as joint angles and velocities, rather than all sensory data^1,61^. These states, combined with system parameters and motion equations, provide the necessary information for predicting future movement (see Supplementary Information Appendix, Eq. S10). It is assumed that the neural controller compares actual joint motion and velocity with the desired and adjusts commands based on their difference. The balance control system’s goal is assumed to maintain a steady, upright stance (equilibrium point), where zero angular motion and velocity ($\dot{\varphi }(t)=\varphi (t)=0$, with $\varphi (t)=\theta (t)-\pi /2$, see Fig. 1) is the target^40,62^. This upright posture allows for maintaining equilibrium and quick responses to perturbations. This study takes upright stance as the reference position and investigates how control strategies adapt to continuous underfloor platform perturbations.

### Controller: neural command centre

With two-thirds of body weight in the upper body, humans are inherently unstable and require continuous control to detect joint deviations from an upright posture and generate corrective motor commands. Understanding these neural commands is challenging due to the complexity of the biological motor control system. In our model’s dynamics equation (see Supplementary Information Appendix, S2), joint torques function as neural commands, regulating joint recovery. To capture the time-varying dynamics of our platform and the self-organised nature of postural strategies, we introduced an MPC framework to simulate the neural command centre’s role in maintaining balance.

The human body maintains balance by continuously processing sensory input. Similarly, an MPC controller uses states information (angular motion and velocity) to assess the actual or current state of the body and compares it with the desired upright stance, as Fig. 1). It then predicts future states and adjusts errors by generating optimal corrective control actions. The MPC calculates corrective commands ($\tau$) by optimising the plant’s output through a cost function over short time intervals. We use a discrete-time state-space model to simplify the design. Since biological systems have limitations, we incorporate biomechanical constraints into a non-linear constrained MPC framework to capture human-like recovery motion^47,54,57,63^. Further details are in the Supplementary Information Appendix.

The non-linear (quadratic) cost function *J* that reflects the control objective is defined as:

1 $$\begin{aligned} J = {({R_s} - Y)^T}\bar{Q}({R_s} - Y) + {(\Delta \Gamma )^T}\bar{R}(\Delta \Gamma ) \end{aligned}$$

The first component of cost function *J* minimises the error between predictions (*Y*) and desired posture ($R_s$), and the second component minimises control action $\Delta \Gamma$, collectively aiming to minimise *J*. The matrices $\bar{R}$ and $\bar{Q}$ are used to tune the closed-loop balance control performance. $\bar{R}=0$ ignores $\Delta \Gamma$’s magnitude, focusing on minimising performance error ${({R_s} - Y)^T}\bar{Q}({R_s} - Y)$. Conversely, high $\bar{R}$ values prioritise controlling $\Delta \Gamma$’s size while cautiously reducing the error ${({R_s} - Y)^T}\bar{Q}({R_s} - Y)$.

The cost function *J* (Eq. 1) uses $3\times 3$ $\bar{R}$ and $6 \times 6$ $\bar{Q}$ matrices as performance tuning parameters, weighting state and control errors. Matrices $\bar{R}$ and $\bar{Q}$ contain a total of 27 independent entries (out of 9 entries in $\bar{R}$ and 36 entries in $\bar{Q}$, due to symmetry $\bar{Q}_{ij}=\bar{Q}_{ji}$, $\bar{R}_{ij}=\bar{R}_{ji}$ for all *i* and *j*), which prove difficult to specify based on either theoretical considerations or experimental data. For simplicity and interpretability of the cost function, $\bar{R}$ and $\bar{Q}$ are assumed diagonal. Each diagonal element represents the weight of its respective parameter.

To replicate experimental conditions in the body dynamics model, we applied the same platform motion, linearly increasing the frequency from 0.4 Hz to 6 Hz while keeping the amplitude constant at 2 mm. MATLAB’s ‘fmincon’ function was used to optimise the cost function *J* (Eq. 1) and determine optimal joint torques for stability. A prediction horizon of Np = 4 and a control horizon of Nc = 2 were selected to achieve effective control performance within system constraints. The sampling frequency was set to 100 Hz.

**Fig. 2:**
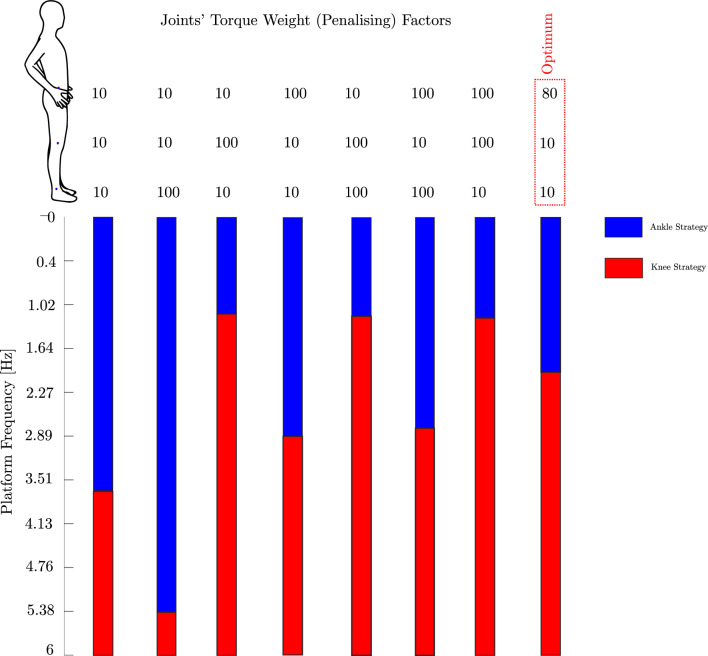
Variation of joint torque weight factors $\bar{R}_{11}$, $\bar{R}_{22}$, and $\bar{R}_{33}$, corresponding to penalties for ankle, knee, and hip torques, and their influence on predicted balance strategies and transition frequencies.

### Identification of postural strategies and coordination transitions

To investigate how COP-COM relationships and postural strategies adapt to changing platform frequencies, we examine joint kinematics, kinetics, and COP-COM relative motion within the MPC framework. These strategies have been assessed against the balance performance of nine (five males and four females, mean age: $26.1 \pm 4.7$ years, mass: $68.4 \pm 5.4$ kg; height: $1.68 \pm 0.07$ m) healthy participants on a moving platform, as detailed in our previous work^15^. In the experiment, participants stood on a 3.6 m by 3.6 m platform at the Exeter VSimulator facility^64^, which allows controlled anterior-posterior floor perturbations. Kinematic and kinetic data were collected using a motion capture system and embedded force plates while participants focused on a visual target. The platform moved sinusoidally, with frequency increasing from 0.4 Hz to 6 Hz at a constant peak amplitude of 2 mm.

Initially, phase relationship plots were visually inspected to detect changes in the phase relationships, particularly the orientations of the phase plots, for adjacent joint torques and COP-COM relative motion. Postural strategies were subsequently identified by analysing the relative phase angles between adjacent joint torques, computed using the vector coding method ^15^. An ankle strategy is defined by in-phase torques of both joint pairs (ankle-knee and knee-hip). A knee strategy is defined by anti-phase torque between ankle-knee and in-phase torque between knee-hip, while a hip strategy is defined by the reverse. A combined strategy is defined by anti-phase torque in both pairs ^65^. The relative phase angles analysed in 1-second windows (100 samples) to determine the proportion of in-phase (blue) and anti-phase (red) coordination between joint pairs (ankle-knee, knee-hip) or between COP and COM in each window. These proportions are then visualised in the relative phase rate (RPR) graph (Fig. 7c–e), where blue and red bars indicate proportion of in-phase and anti-phase patterns, respectively. The RPR graph illustrates how coordination evolves with platform frequency, with strategy transitions identified by shifts in the dominant coordination pattern over time.

## Results

### Influence of performance and effort penalties on balance strategy transitions

The weighting matrices $\bar{Q}$ and $\bar{R}$ were adjusted to balance the trade-off between deviations from a stable upright posture and control effort. We discovered that adjustments to $\bar{Q}$ had minimal impact, while varying $\bar{R}$ significantly influenced balance performance. Consequently, $\bar{Q}$ was set as a constant diagonal matrix, an identity matrix scaled by 10 (Eq. 2), implying equal weighting for all states, including both joint angular motions and velocities. We then investigated how varying $\bar{R}$, the joint torque weights, impacts the performance of our MPC scheme. Figure 2 shows the effect of adjusting joint torque weight factors on balance control strategies and transition frequencies. Different torque weighting configurations within the MPC model effectively maintained balance during time-varying platform motions, indicating the flexibility of the model to simulate diverse individual behaviours and priorities. Equal torque cost weighting at the ankle, knee, and hip joints resulted in a transition from an ankle to knee strategy at 3.65 Hz, consistent with the behaviour of participants with regular athletic training, who transitioned at frequencies above the average of 2.11 Hz. To align the model’s ankle-to-knee strategy transitions with the experimental average frequency of 2.11 Hz, specific weights for $\bar{Q}$ and $\bar{R}$ were chosen. The state weight $\bar{Q}$ and input weight $\bar{R}$ were selected as follows:

2 $$\begin{aligned} \begin{aligned} \bar{R}&= \begin{bmatrix} r_{11} & 0 & 0 \\ 0 & r_{22} & 0 \\ 0 & 0 & r_{33} \\ \end{bmatrix} = \begin{bmatrix} 10 & 0 & 0 \\ 0 & 10 & 0 \\ 0 & 0 & 80 \\ \end{bmatrix}, \quad \\ \bar{Q}&= \begin{bmatrix} q_{11} & 0 & 0 & 0 & 0 & 0 \\ 0 & q_{22} & 0 & 0 & 0 & 0 \\ 0 & 0 & q_{33} & 0 & 0 & 0 \\ 0 & 0 & 0 & q_{44} & 0 & 0 \\ 0 & 0 & 0 & 0 & q_{55} & 0 \\ 0 & 0 & 0 & 0 & 0 & q_{66} \\ \end{bmatrix} = 10 \times I_{6\times 6} \end{aligned} \end{aligned}$$

### Phase transition plots

**Fig. 3:**
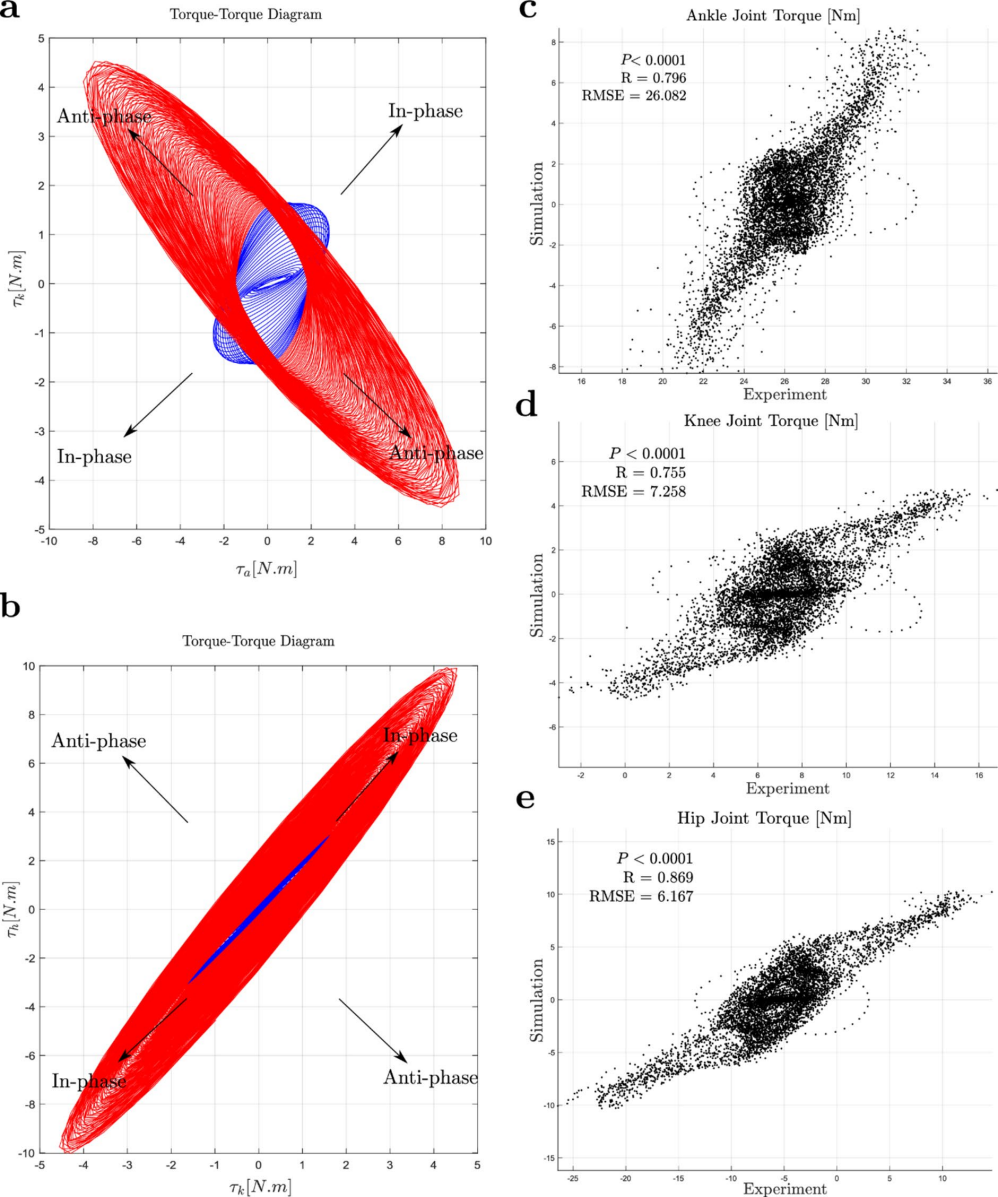
Joint torque phase plots and simulation-experiment comparison: (**a, b**) Torque-torque phase plots showing how ankle-knee and knee-hip joint torques adjust to increasing perturbation frequency, respectively. The phase trajectories progress outward from the centre as the platform frequency increases from 0.4 Hz to 6 Hz. Blue cycles correspond to lower frequencies (up to approximately 2.11 Hz) and indicate an ankle strategy (in-phase torque between ankle-knee and knee-hip joints). Red cycles correspond to higher frequencies (above  2.11 Hz) and represent a knee strategy (in-phase knee-hip torque accompanied by anti-phase ankle-knee torque). The transition between strategies is marked by a shift in the ankle-knee torque from in-phase to anti-phase, while knee-hip torque remains consistently in-phase. (**c–e**) Comparison between simulated and experimental joint torques for the ankle, knee, and hip. Strong correlations are observed across all joints ($P < 0.0001$), with the hip showing the highest agreement ($R = 0.869$, RMSE = 6.167), followed by the ankle ($R = 0.796$, RMSE = 26.082) and knee ($R = 0.755$, RMSE = 7.258). These comparisons support the model’s ability to reproduce joint-level torque behaviours observed in experimental data.

Figure 3a, b illustrates the evolving coordination between ankle-knee and knee-hip joint torques as the perturbation frequency increases from 0.4 to 6 Hz. Changes in coordination are captured through shifts in the orientation and progression of the phase trajectories. An in-phase relationship between ankle-knee and knee-hip torques (blue cycles oriented toward 45/225° in both plots) corresponds to an ankle strategy. In contrast, a knee strategy is indicated by an in-phase relationship between knee-hip torques (red cycles at 45/225° in Fig. 3b and an anti-phase relationship between ankle-knee torques (red cycles at 135/315° in Fig. 3a). As the perturbation frequency increases, the plot begins at the central inner cycles (representing lower frequencies) and moves outward, with joint torque magnitudes increasing accordingly to compensate for faster perturbations. The key feature of the plot is the transition in ankle-knee torque orientation, transitioning from 45/225° (in-phase) to 135/315° (anti-phase). In contrast, the knee-hip torque maintains a consistent in-phase relationship, with cycles oriented at 45/225° throughout. This transitions marks a shift from an ankle strategy to a knee strategy which likely aimed at avoiding excessive ankle torque without lifting the heels^15,62^.

To assess how well the MPC model replicates joint-level dynamics, we compared simulated and experimental torques at the ankle, knee, and hip (Fig. 3c–e). Pearson correlation (*R*) and RMSE were used to assess linear agreement and deviation magnitude. All joints showed significant correlations with experimental data ($P < 0.0001$), indicating the model captures key torque dynamics. The hip showed the strongest match ($R = 0.869$, RMSE = 6.167 Nm), followed by ankle ($R = 0.796$, RMSE = 26.082 Nm) and knee ($R = 0.755$, RMSE = 7.258 Nm). High RMSE values were consistent with the average experimental torques (see Fig. 3c–e), suggesting the error reflects a systematic offset rather than tracking inaccuracy. This offset likely results from modelling upright standing as a zero-reference posture, whereas experimental torques include baseline loading and subject-specific postural biases. This effect is most pronounced at the ankle, which contributes most to static balance and typically exhibits higher baseline torque.

**Fig. 4:**
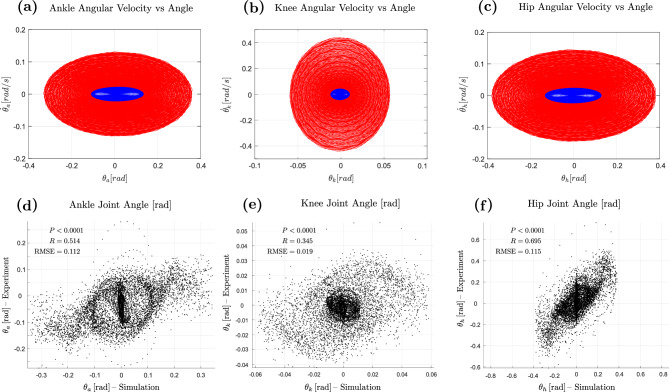
Angular motion-velocity phase plot and joint angle simulation-experiment comparison: (**a–c**) as perturbation frequency increases, joint recovery motions become faster and larger. In these phase plots, blue cycles correspond to lower frequencies (up to approximately 2.11 Hz) and reflect an ankle strategy, whereas red cycles correspond to higher frequencies (above $\sim$2.11 Hz) and reflect a knee strategy. The knee joint shows a bias towards higher angular velocity, while the ankle and hip joints exhibit greater angular motion. (**d–f**) Comparison between simulated and experimental joint angles for the ankle, knee, and hip. Significant correlations were observed across joints ($P < 0.0001$), with the hip showing the highest agreement ($R = 0.695$, RMSE = 0.115 rad), followed by the ankle ($R = 0.514$, RMSE = 0.112 rad) and knee ($R = 0.345$, RMSE = 0.019 rad).

Figure 4a–c depicts the relationship between joint angular motion and velocity at the ankle, knee, and hip during recovery from perturbations. The blue and red cycles corresponds to the ankle and knee strategy, respectively, as seen in Fig. 3. The knee joint is more biased towards angular velocity, while the ankle and hip are more oriented towards angular motion. This shows that the knee joint generates greater angular velocity, but less angular motion compared to the other joints, emphasising its crucial role in balance recovery during high-frequency perturbations.

We further evaluated the MPC model’s ability to reproduce joint angles at the ankle, knee, and hip (Fig. 4d–f). All correlations were statistically significant ($P < 0.0001$). The hip showed the strongest agreement ($R = 0.695$, RMSE = 0.115 rad), followed by the ankle ($R = 0.514$, RMSE = 0.112 rad) and knee ($R = 0.345$, RMSE = 0.019 rad). The lower *R* values for joint angles are likely because angles are indirectly generated from the torques applied by the MPC model. Since torques serve as inputs to a simplified body dynamics model (plant), additional modelling errors are introduced that are not present in the directly controlled torque outputs. The knee’s relatively lower *R* may also result from its intermediate role, where errors at the ankle or hip influence its motion. Despite this, the model captures key angular trends, especially at the hip and ankle.

**Fig. 5:**
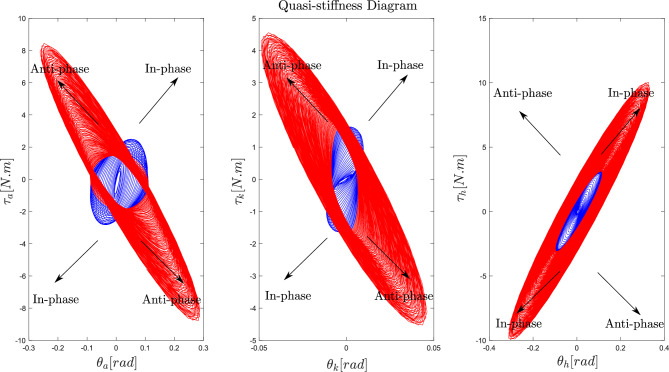
Torque–angle phase plot: this plot demonstrates the quasi-stiffness of the ankle, knee, and hip joints as a function of platform frequency. In these phase plots, blue cycles represent joint quasi-stiffness under an ankle strategy at lower platform frequencies (up to approximately 2.11 Hz), while red cycles reflect quasi-stiffness at higher frequencies (above $\sim$2.11 Hz), corresponding to a knee strategy. The slope of each curve represents the effective stiffness of the joint and shows into how joint angles and torques are coordinated. The transition in ankle and knee joint quasi-stiffness sign corresponds to the transition from an ankle to a knee strategy at higher frequencies.

Figure 5 illustrates torque-angle plots capturing the quasi-stiffness, which represents the relationship between joint torques and angles during backward and forward sway cycles while standing on the sinusoidally moving platform. Quasi-stiffness, often defined as the slope of the best linear fit on the torque-angle graph, reflects the apparent stiffness of a joint throughout the movement cycle. The ankle and knee joints (first and second plots from the left) exhibit a transition from an in-phase relationship (blue cycles) to an anti-phase relationship (red cycles) as the perturbation frequency increases. In contrast, the hip joint maintains an in-phase relationship between joint angle and torque across all frequencies. This transition, marked by a change in the slope of the quasi-stiffness graph from positive to negative for the ankle and knee joints, corresponds to shift from an ankle strategy to a knee strategy.

**Fig. 6:**
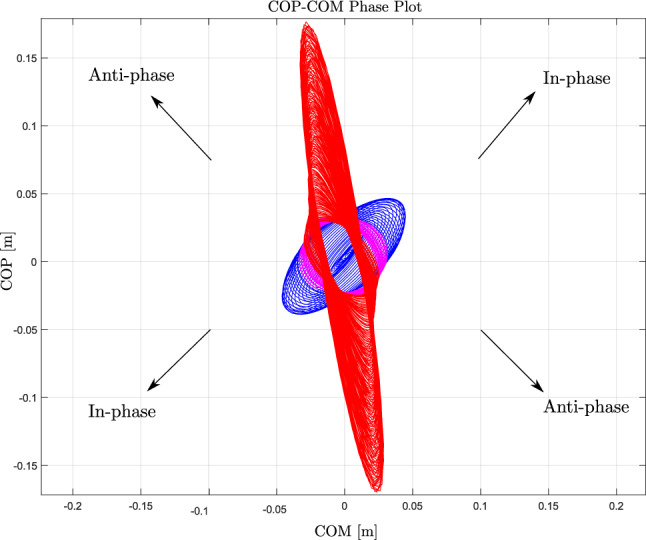
COP-COM phase plot: this plot illustrates the relative motion of the COP and COM as platform frequency increases. The transition from in-phase (blue cycles) to anti-phase (red cycles) motion corresponds to the shift from an ankle strategy to a knee strategy. Pink cycles indicate a period of instability just before the transition.

The phase plot in Fig. 6 shows the transition from an in-phase (blue cycles) to an anti-phase (red cycles) relationship between COP and COM motion as platform frequency increases. This transition corresponds to the transition from ankle to knee strategy. At lower frequencies, the phase plot (starting from the origin) indicates an in-phase relationship between COM and COM with the dominance of COP motion. However, as the platform’s frequency increases, COM gets larger and gains dominancy over COP. As shown in the Fig. 6, the cycles have a bias towards COM axis just before transitions from blue to red cycles, signified by pink cycles and indicating an unstable balance control. At this point, by bending the knee and transitioning to a knee strategy, the COP becomes anti-phase with the COM and regains its dominancy. This dominancy shift can be seen by the bias or stretch of the phase plot towards COP motion before and after postural strategy transition, which is indicative of a stable situation. Specifically, during high frequency, the red graphs are more biased to the COP axis which indicate a more stable balance control with knee strategy.

### Transition between balance control strategies

**Fig. 7:**
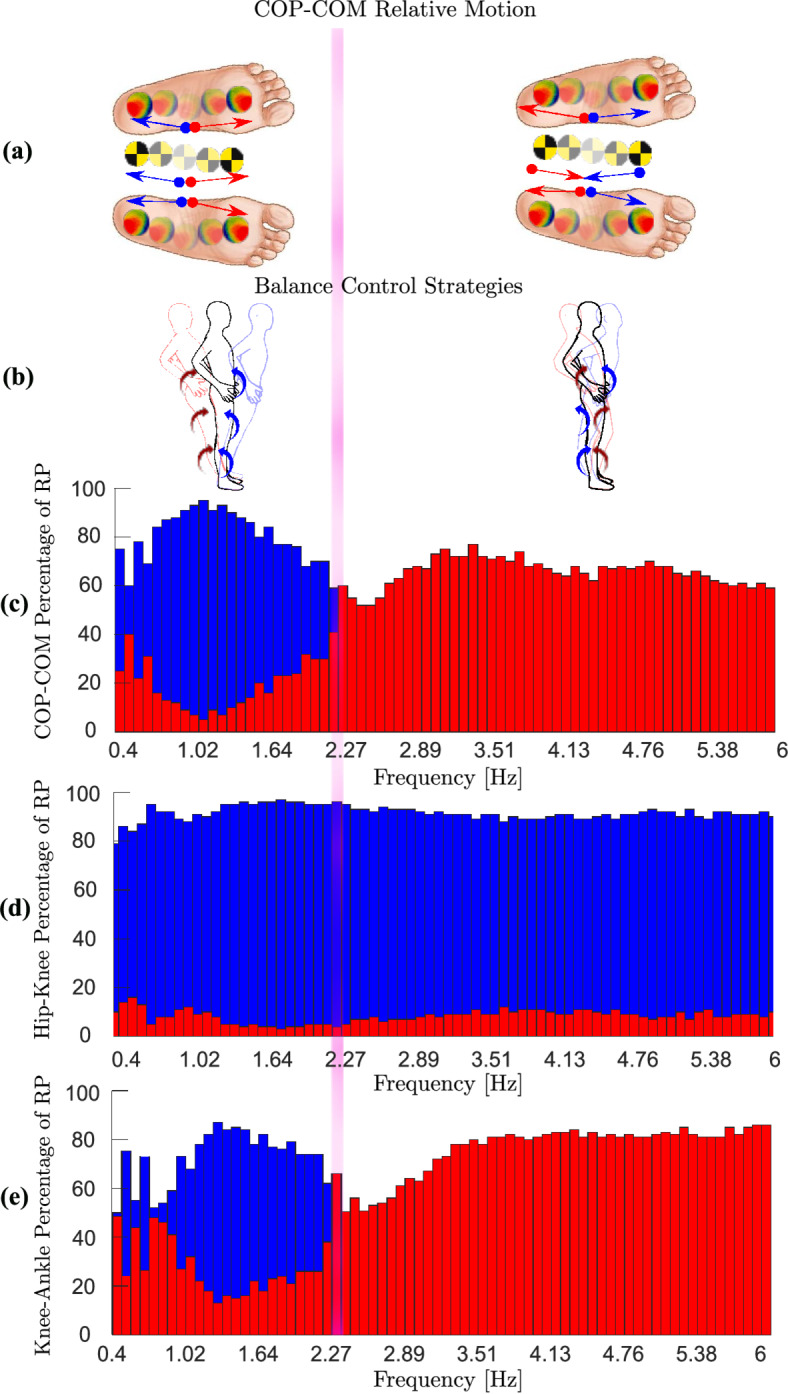
Transitions in relative phase: (**a**) COP-COM relative motion, with forward (FW) and backward (BW) motion shown by red and blue arrows, respectively. (**b**) Transition between balance control strategies as platform frequency increases. (**c–e**) Relative phase rate (RPR) graphs for COP-COM (**c**), hip-knee torque (**d**), and knee-ankle torque (**e**) as a function of platform frequency. Each bar in panels (**c–e**) represents the proportion of in-phase (blue) versus anti-phase (red) coordination within a 1-s window. The observed shift from blue to red bars reflects transitions in coordination dynamics, marking a change in balance control strategy.

We used the RPR graph (Fig.7c–e), as described in the Methods and in^15^, to identify transition frequencies. As the platform frequency increased from 0.4 to 6Hz, we observed a shift from blue to red bars in Fig. 7c, e, indicating a transition from in-phase to anti-phase coordination in the ankle-knee and COP-COM relationships within the 2.19-2.27Hz window. In contrast, the hip-knee pair remained predominantly in-phase. This pattern, illustrated schematically in Fig.7a, b, indicates a shift from an ankle to a knee strategy and a reorganisation of COP-COM coordination. The exact transition frequencies—2.14 Hz for knee-ankle and 2.24 Hz for COP-COM—were estimated using a sigmoid fitting method ^15^.

These model-derived frequencies align with our experimental findings ^15^, where mean transition frequencies across participants were $2.11 \pm 0.11\,\text {Hz}$ for knee-ankle and $2.26 \pm 0.18\,\text {Hz}$ for COP-COM coordination. This difference in the experimental data was statistically significant (paired t-test, $p < 0.001$), suggesting that joint-level changes precede global COP-COM adjustments, a recognised global variable ^14^. This supports a hierarchical adaptation process: transitions in local strategies emerge first, followed by delayed global reorganisation in COP-COM relationship to restore balance.

**Fig. 8:**
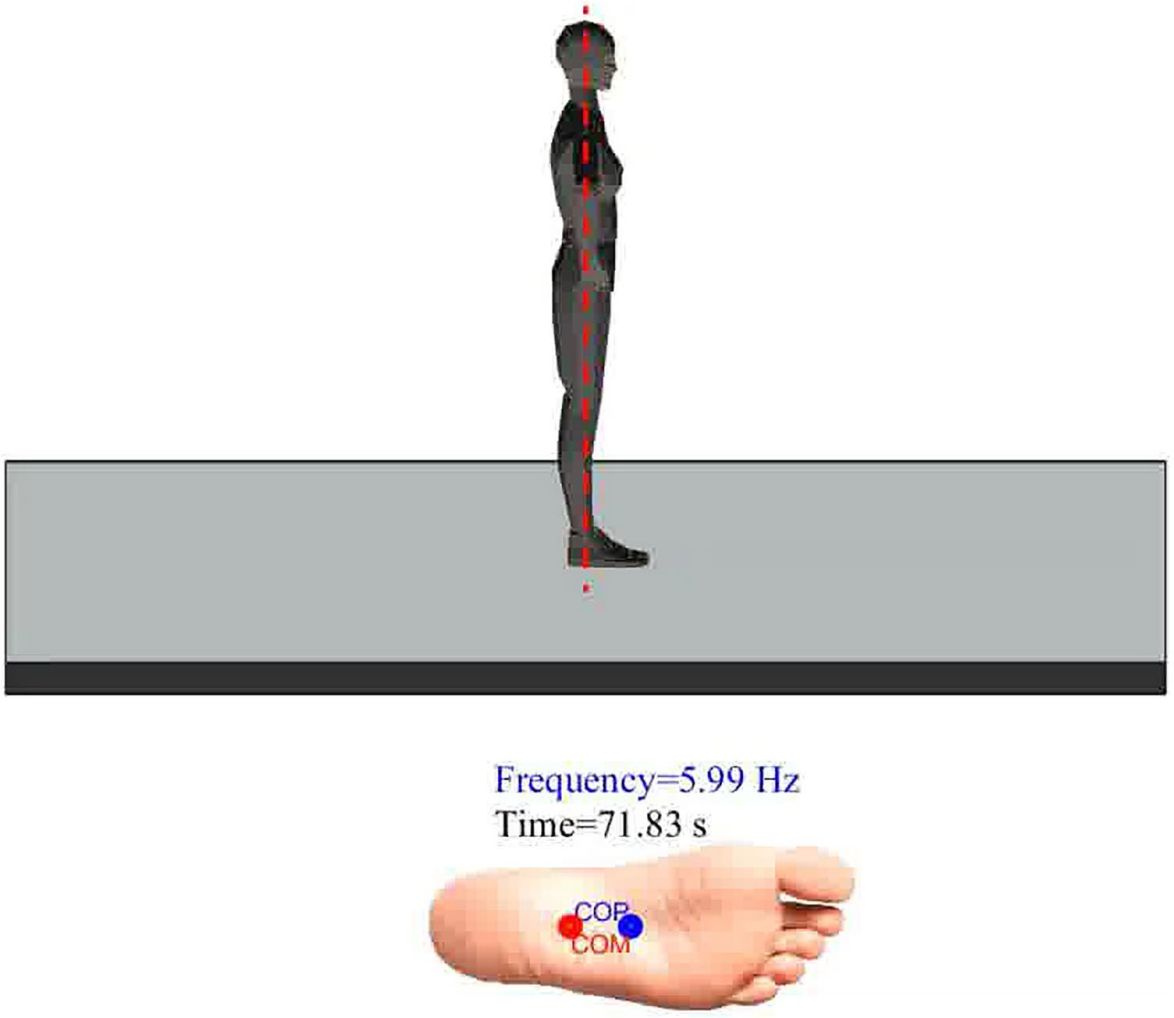
The animation shows the interaction of a human model with the moving platform as platform frequency increases. The red and blue markers represent the COM and COP, respectively, while the red line indicates the equilibrium point or desired postural position (see also Supplementary Movie S1 in the Supplementary Information, animation version is published as Electronic supplementary material).

A MATLAB model was developed to visually present the simulation results, as shown in Fig. 8. In this figure, the red and blue markers represent the COM and COP, respectively, while the red line denotes the equilibrium point or desired position for the postural control system. The animated model demonstrates the transition between postural strategies and the COP-COM relative motion and is available as a Supplementary Movie S1. This model builds on an articulated humanoid body originally derived from a VRML file^66^, later converted into a MATLAB file by Tordoff and Mayol from the Robotics Research Group at the University of Oxford^67^. We adapted this static model to reflect the dynamic postural response of a humanoid standing on a moving platform, with hands on the hips, as per our experimental setup, and incorporated COP-COM relative motion. To simulate realistic dynamic motion, we animated the human model with our MPC output motion. To better visualise platform movement, we magnified the platform’s motion and slowed down the output to display the relatively small (2 mm) platform movement and fast frequencies (up to 6 Hz). The video includes the real frequency and timing of the platform’s motion.

## Discussion

This study introduces a novel MPC framework for simulating human balance control responses to continuous and time-varying translational perturbations. The plant model, purely based on body dynamics, does not integrate experimental data and uses a fixed parameter set across perturbation frequencies. Despite this, the model adapted to frequency changes and optimised balance strategies (performance and effort costs) within biomechanical constraints. The model predicts stability loss resulting from transitions in COP motion dominance over COM motion. Consistent with the self-organising nature of postural control, defined as the spontaneous emergence of new coordination patterns in response to gradual changes in a control parameter (platform frequency), we observed a shift from in-phase to anti-phase COP-COM motion and a transition from ankle to knee strategy as frequency increased.

### Adaptive and robust selection of balance control strategies: the self-organised nature of postural strategies

Our study provides a step-change in understanding of how the body adapts to maintain balance under time-varying underfloor perturbation. Our model demonstrates robustness and adaptability to continuous, time-varying perturbation. It restores balance using various parameter sets and self-organised optimal balance control strategies without adjusting parameters over time and autonomously transitioned between them, as depicted in Fig. 2. This aligns with our experimental observations^15^ and prior experimental research based on self-organisation principals^14,17,20–22^, indicating that the non-linear transitions between postural strategies emerge naturally from the interactions of various components of the body (such as muscles, joints, and the nervous system) with environment without the need for a pre-programmed set of instructions in the brain. To capture available balance control strategies, such as ankle and hip strategies, prior modelling studies have suggested a switching mechanism between controllers at specific thresholds. For example,^68,69^ employed two decoupled or separate controllers, while^70^ proposed a classifier to individually select and optimise each strategy based on specific objectives and constraint satisfaction. Park et al.^40^ designed separate LQRs for each perturbation condition, adapting the optimisation criterion to capture different strategies based on perturbation magnitude. Atkeson and Stephens^71^ introduced a unified criterion using a quadratic cost function, effectively generating multiple balance recovery strategies, including ankle and hip strategies, across various perturbation magnitudes. However, these models primarily addressed short, discrete perturbations in specific directions, with the transition between strategies triggered by thresholding or saturation methods. In other words, strategy selection is based on predefined conditions rather than the system self-organising as seen in our study, eliminating the need for manual tuning^62,72^. The proposed MPC framework offers a more flexible and adaptive approach compared to traditional control methods such as LQR and PID, which are designed for time-invariant systems. Traditional controllers typically separate planning and execution, leading to limited adaptability in time-varying scenarios. In contrast, an MPC framework continuously updates control actions by solving an optimisation problem at each time step, allowing simultaneous planning and execution. This predictive capability makes MPC inherently more flexible and responsive to changing conditions, which is crucial for modelling balance control in response to time-varying perturbations. LQR controllers require distinct set of gain matrices for varying perturbation condition, as shown by^40,62^ for different perturbation magnitudes; however, MPC framework can adapt dynamically, making it better suited for capturing human responses to unpredictable environmental changes. Previous MPC works, such as those by Aftab et al.^47,73^ and Shen et al.^54^, applied MPC framework to balance control but primarily focused on discrete, short or time-invariant disturbances. Our MPC framework significantly advances these models by addressing continuous, dynamic perturbations, better reflecting the complexities of real-world balance control. This adaptability not only overcomes the limitations of traditional control methods but also reveals new insights into how the body maintains balance in the face of dynamic, time-varying disturbances in real-world scenario.

### The knee joint role

Our previous experiments^15^ were the first to identify the knee strategy as a relevant postural control strategy in real-world settings, revealing the transition between ankle and knee strategies that underpins the shift in COP-COM relative motion. Consistent with these experimental findings, the model presented here also shows a transition from ankle to knee strategy in response to high-frequency, small-amplitude platform motions, which more closely reflect real-world conditions. Recent studies, such as Jones et al.^74^ and Wan et al.^75^, further highlight the importance of knee involvement in balance strategies. Jones et al. demonstrated that while the ankle strategy suffices for low-amplitude perturbations (up to 10 mm), higher amplitudes require more frequent use of hip and knee strategies for older subjects. Similarly, Wan et al. showed that knee torque plays a crucial role in balance recovery through direction-specific feedback gains, emphasising the knee’s significance in developing assistive technologies. Our study builds on these findings and challenges earlier views, such as those by Shen et al.^63^, who suggested that the knee joint is only engaged during large perturbations. While our results confirm that small-amplitude perturbations produce smaller knee angular motion compared to the ankle and hip, the knee still plays a significant role at higher platform frequencies, bending with high angular velocity and torque. This indicates the knee strategy’s importance in dynamic balance control at higher frequencies. Future models should integrate knee motion to more accurately represent human balance control in real-world conditions.

### Selection of states and torque objectives

We used a generalised cost function that combines effort-related and performance-related objectives through a weighted summation. The neural command centre was assumed to generate optimal corrective torques to stabilize the body in an upright position. By setting the weighting matrices $\bar{R}$ and $\bar{Q}$, we minimised corrective torque and performance errors. Empirical observations showed that varying the *Q* matrix, while keeping *R* constant, had minimal impact on balance recovery, suggesting that for small perturbations, joint angular deviations and velocities are equally weighted. Thus, optimising the overall cost function *J* by adjusting $\bar{R}$, which minimises joint torque effort, may be more effective than focusing on minor joint angle deviations. Furthermore, simulation results indicate that placing a greater penalty on hip joint corrective torques, compared to knee and ankle joints, aligns with previous experimental findings^15^. This suggests that minimising hip torque is preferred in healthy young individuals, possibly due to biomechanical factors or inefficiencies in using the hip for balance recovery during small-amplitude perturbations.

### Changes in quasi-stiffness under effect of platform frequency

Our findings demonstrate a consistent scaling pattern in the torque-angle cycle size across all joints as frequency increases, as shown in Fig. 5. The ankle and knee joints transition from positive to negative tangential slopes in the quasi-stiffness graph with increasing frequency, while the hip joint maintains a positive slope at all frequencies. A positive slope indicates an in-phase, spring-like behaviour where torque increases with joint movement, whereas a negative slope at the ankle and knee suggests an anti-phase relationship, where these joints may assist movement rather than resist it. This transition reflects a reorganisation of coordination patterns and may indicate changes in muscle activation or neural control strategies as the system adapts to maintain stability at higher frequencies.

At low frequencies, the positive quasi-stiffness of the ankle strategy generates torque to counteract joint angle deviations, restoring upright equilibrium position. In contrast, at higher frequencies, the negative quasi-stiffness of the knee strategy destabilises the joints, moving the shank and thigh segments away from the equilibrium. Interestingly, despite this destabilising effect, overall system stability improves due to the knee strategy. The cost function in our study (Eq. 1) balances deviations from upright equilibrium (joint motion costs) with minimising control effort (torque costs).

At higher frequencies, employing the knee strategy involves greater knee flexion and larger joint angle variations, as indicated by the larger red cycles compared to the blue cycles in Fig. 5. While this might appear destabilising, the anti-phase torque generated mitigates the sharp increase in joint torque seen in the ankle strategy. Thus, the CNS seems to prioritise minimising torque costs over small joint motion deviations at higher frequencies. Even though the perturbation amplitude remains small, the forces and accelerations increase with platform frequency, making torque reduction an efficient strategy for the CNS in handling these high-frequency perturbations.

Our findings reveal a transition in the tangential slope of the $\tau -\theta$ graph as platform frequency increases, a dynamic that traditional control systems such as LQR and PID may not capture. LQR controllers typically result in a consistently negative tangential slope in the quasi-stiffness graph, which does not reflect the transitional dynamics observed in our study. It is important to differentiate between quasi-stiffness^76,77^ and the stiffness used in mass-spring-damper models^78–80^. Quasi-stiffness can be both positive and negative, while genuine stiffness is always positive^81,82^. Cerda et al.^83^ and Rouse et al.^82^ showed that quasi-stiffness sign can vary during movement, reflecting adaptive control strategies. This variation is crucial for capturing the dynamic nature of joint mechanics. In our model, this transition in quasi-stiffness sign occurred naturally due to the system’s adaptation to perturbation conditions. This inherent adaptability better simulates human joint behaviour during balance recovery, where passive stiffness alone cannot fully explain the system’s response.

Predicted joint torques from our MPC model showed stronger agreement with experimental data than joint angles. This is likely because torque is the primary output of the MPC and explicitly optimised over time. Prior musculoskeletal simulations under perturbation conditions reported lower agreement for joint angles and torques. Koelewijn and Ijspeert ^43^ found lower torque correlations and moderate angle agreement using reflex-based control, while Shanbhag et al. ^42^ reproduced motion trajectories generally comparable to experimental data. These differences may reflect variations in control formulation: in the musculoskeletal simulations, torques emerge from feedback-driven muscle activations and can be more difficult to match directly. Strong torque agreement is critical in our study, as postural strategies are identified through torque coordination. In contrast, joint angles depend on how torques interact with our body dynamics model (plant) and are more sensitive to simplifications in body dynamics. Our model also assumes full-state feedback and omits noise which reduces variability in output joint motion.

Finally, the goal of our model is to maintain stability with minimal effort, unlike prior studies^40,62^, which focused on minimising errors between model states and experimental data. Although the exact optimisation strategy of the CNS remains unclear, our cost function is designed to prioritise minimal deviations from upright stance with minimal exertion, rather than reducing errors relative to an external dataset. This approach is more consistent with how the CNS might maintain balance, focusing on limiting body motion with minimal torque or effort.

## Limitations and future works

In this study, we incorporated time-varying perturbations to gain deeper insights into human balance in the face of real-world complexities, compared to traditional discrete, time-invariant perturbations. However, the perturbations in this study are still relatively simple compared to the dynamic, complex scenarios encountered in real environments. Despite this, our MPC-based model provides a strong foundation for future research on more realistic and dynamic postural responses. The model can be extended to account for more complex perturbations. For instance, while MPCs typically assume accurate state information at any given time, real-world conditions often involve sensory noise and incomplete information, making it difficult for the controller to estimate the system’s state accurately. Future work will address this by incorporating a ‘state observer’ (e.g., a Kalman filter) to estimate system states and account for sensory signal noise.

Kim et al.^62^ found that the CNS adjusts joint torques to accommodate biomechanical constraints across different populations: young adults, older adults, and individuals with Parkinson’s disease. The study observed that older adults had smaller ankle movements and torques compared to younger individuals but showed slightly larger hip movements and torques. In contrast, Parkinson’s patients displayed significantly reduced hip movement and torque compared to older adults, particularly during moderate perturbations. This suggests that older adults rely more on hip movements for balance, while Parkinson’s patients have further impaired hip responses. Our model can simulate different populations’ behaviour by adjusting penalties on joint-specific torques and angles. For this study, we used fixed parameters to replicate young adults’ behaviour in our experiment, ensuring the ankle-to-knee strategy transition close to the observed frequency of 2.11 Hz. Future work can adjust these penalties to simulate other populations- behaviour in their experiments.

We simplified the selection of the diagonal elements for matrices $\bar{Q}$ and $\bar{R}$ to replicate our experimental observation in terms of transition frequency and balance control strategies. Future studies could develop a parametrised formula for these matrices in a physically meaningful way as suggested in^26^, allowing a broader exploration of balance control strategies. Given the challenge of iteratively determining all parameter combinations, such a formula could offer a more efficient approach.

In our experiment, participants maintained balance without stepping, consistent with our model’s focus on replicating non-stepping fixed support strategies. However, stepping is often employed in response to larger perturbations. A key extension of our model would be the integration of stepping strategies, enabling simulations of scenarios where non-stepping balance strategies alone are insufficient.

## Conclusion

We present a four-segment body dynamic model based on MPC to emulate human balance responses to continuous time-varying perturbations. Unlike previous models, our model does not rely on predefined conditions for balance control strategies recruitment. Despite this, the model autonomously recruit a new postural strategy to deal with changes in underfloor platform speed. The model shows that at lower frequencies, the COP and COM move in-phase, with the COP leading, accompanied by an ankle strategy where the body acts like an inverted pendulum. As platform frequency increases, the system becomes mechanically unstable, prompting a transition to a knee strategy, where COP and COM move in anti-phase, restoring COP dominance and stabilising the system. Unlike the ankle strategy, the knee strategy remains stable up to 6 Hz, demonstrating its greater robustness. Our MPC framework maintained balance across platform frequencies without data-driven inputs, replicating key features of human balance observed in experiments. The results suggest that effective models of human posture should include ankle, knee, and hip movements. Additionally, placing a higher penalty on hip joint torques effectively simulates balance in healthy young individuals, likely due to biomechanical constraints that make the hip less efficient for small perturbations. The model also provides new insights into the transition from ankle to knee strategies, as seen in the simultaneous change in quasi-stiffness slopes from positive to negative. This distinction is crucial for designing biomimetic control systems for powered prosthetics, as balance control models with fixed feedback gains may not fully capture transitional dynamics observed in experimental studies.

## Supplementary Information

Supplementary Video 1.

Supplementary Information.

## Data Availability

The experimental datasets used and/or analysed during the current study are available from the corresponding author upon reasonable request in an online repository at: https://ore.exeter.ac.uk/repository/handle/10871/131883.

## References

[1] Horak, F. B. & Macpherson, J. M. *Handbook of Physiology, Section 12: Exercise: Regulation and Integration of Multiple Systems* (American Physiological Society, 1996).

[2] Maurer, C., Mergner, T. & Peterka, R. Multisensory control of human upright stance. *Exp. Brain Res.* **171**, 231–250 (2006). 16307252 10.1007/s00221-005-0256-y

[3] Schmidt, R. & Lee, T. Motor Control and Learning: Human Kinetics (1988).

[4] Latash, M. Evolution of motor control: From reflexes and motor programs to the equilibrium-point hypothesis. *J. Hum. Kinet.* **19**, 3–24 (2008). 19823595 10.2478/v10078-008-0001-2 PMC2759721

[5] Towhidkhah, F., Gander, R. & Wood, H. Model predictive impedance control: A model for joint movement. *Journal of motor behavior* **29**, 209–222 (1997). 12453780 10.1080/00222899709600836

[6] Horak, F. B. & Nashner, L. M. Central programming of postural movements: Adaptation to altered support-surface configurations. *J. Neurophysiol.* **55**, 1369–1381 (1986). 3734861 10.1152/jn.1986.55.6.1369

[7] Keshner, E., Woollacott, M. & Debu, B. Neck, trunk and limb muscle responses during postural perturbations in humans. *Exp. Brain Res.* **71**, 455–466 (1988). 3416963 10.1007/BF00248739

[8] Horak, F. B., Henry, S. M. & Shumway-Cook, A. Postural perturbations: New insights for treatment of balance disorders. *Phys. Ther.* **77**, 517–533 (1997). 9149762 10.1093/ptj/77.5.517

[9] Mohseni, O. et al. Balance recovery schemes following mediolateral gyroscopic moment perturbations during walking. *PloS one* **19**, e0315414 (2024). 39739770 10.1371/journal.pone.0315414 PMC11687818

[10] Mohseni, O. et al. Muscular responses to upper body mediolateral angular momentum perturbations during overground walking. *Front. Bioeng. Biotechnol.* **13**, 1509090. 10.3389/fbioe.2025.1509090 PMC12021904 40291562

[11] Xu, X., Bowtell, J., Fong, D. T., Young, W. R. & Williams, G. K. Kinematics of balance controls in people with chronic ankle instability during unilateral stance on a moving platform. *Sci. Rep.* **15**, 1126 (2025). 39774772 10.1038/s41598-025-85220-x PMC11707225

[12] Tarabini, M., Solbiati, S., Moschioni, G., Saggin, B. & Scaccabarozzi, D. Analysis of non-linear response of the human body to vertical whole-body vibration. *Ergonomics* **57**, 1711–1723 (2014). 25105223 10.1080/00140139.2014.945494

[13] Tarabini, M., Saggin, B., Scaccabarozzi, D., Gaviraghi, D. & Moschioni, G. Apparent mass distribution at the feet of standing subjects exposed to whole-body vibration. *Ergonomics* **56**, 842–855 (2013). 23510270 10.1080/00140139.2013.776704

[14] Ko, J.-H., Challis, J. H. & Newell, K. M. Transition of com-cop relative phase in a dynamic balance task. *Hum. Mov. Sci.* **38**, 1–14 (2014). 25240175 10.1016/j.humov.2014.08.005

[15] Taleshi, N., Brownjohn, J. M., Lamb, S. E., Zivanovic, S. & Williams, G. K. Vector coding reveals the underlying balance control strategies used by humans during translational perturbation. *Sci. Rep.* **12**, 21030 (2022). 36470936 10.1038/s41598-022-24731-3 PMC9722668

[16] Yang, J., Winter, D. & Wells, R. Postural dynamics in the standing human. *Biol. Cybern.* **62**, 309–320 (1990). 2310785 10.1007/BF00201445

[17] Bardy, B. G., Marin, L., Stoffregen, T. A. & Bootsma, R. J. Postural coordination modes considered as emergent phenomena. *J. Exp. Psychol. Hum. Percept. Perform.* **25**, 1284 (1999). 10531664 10.1037//0096-1523.25.5.1284

[18] Turvey, M. T. Coordination. *Am. Psychol.* **45**, 938 (1990). 2221565 10.1037//0003-066x.45.8.938

[19] Kelso, J. S. *Dynamic Patterns: The Self-Organization of Brain and Behavior* (MIT Press, 1995).

[20] Dutt-Mazumder, A. & Newell, K. Transitions of postural coordination as a function of frequency of the moving support platform. *Hum. Mov. Sci.* **52**, 24–35 (2017). 28103469 10.1016/j.humov.2017.01.006

[21] Dutt-Mazumder, A., Rand, T. J., Mukherjee, M. & Newell, K. M. Scaling oscillatory platform frequency reveals recurrence of intermittent postural attractor states. *Sci. Rep.* **8**, 1–10 (2018). 30068921 10.1038/s41598-018-29844-2 PMC6070516

[22] Mohd Ramli, N. F. F., Mat Dzahir, M. A. & Yamamoto, S.-I. Estimation of transition frequency during continuous translation surface perturbation. *Appl. Sci.* **9**, 4891 (2019).

[23] Taleshi, N. Human posture control on a dynamic platform. https://ore.exeter.ac.uk/repository/handle/10871/135419. PhD thesis, University of Exeter (2024).

[24] Peterka, R. J. Sensorimotor integration in human postural control. *J. Neurophysiol.* **88**, 1097–1118 (2002). 12205132 10.1152/jn.2002.88.3.1097

[25] Mergner, T., Maurer, C. & Peterka, R. A multisensory posture control model of human upright stance. *Prog. Brain Res.* **142**, 189–201 (2003). 12693262 10.1016/S0079-6123(03)42014-1

[26] Kuo, A. D. An optimal control model for analyzing human postural balance. *IEEE Trans. Biomed. Eng.* **42**, 87–101 (1995). 7851935 10.1109/10.362914

[27] Peterka, R. J. Simplifying the complexities of maintaining balance. *IEEE Eng. Med. Biol. Mag.* **22**, 63–68 (2003). 12733461 10.1109/memb.2003.1195698

[28] Johansson, R., Magnusson, M. & Akesson, M. Identification of human postural dynamics. *IEEE Trans. Biomed. Eng.* **35**, 858–869 (1988). 3192235 10.1109/10.7293

[29] Peterka, R. J. Postural control model interpretation of stabilogram diffusion analysis. *Biol. Cybern.* **82**, 335–343 (2000). 10804065 10.1007/s004220050587

[30] Kiemel, T., Oie, K. S. & Jeka, J. J. Multisensory fusion and the stochastic structure of postural sway. *Biol. Cybern.* **87**, 262–277 (2002). 12386742 10.1007/s00422-002-0333-2

[31] Shanbhag, J. et al. Methods for integrating postural control into biomechanical human simulations: A systematic review. *J. Neuroeng. Rehabil.* **20**, 111 (2023). 37605197 10.1186/s12984-023-01235-3 PMC10440942

[32] Jiang, Y., Nagasaki, S., You, M. & Zhou, J. Dynamic studies on human body sway by using a simple model with special concerns on the pelvic and muscle roles. *Asian J. Control* **8**, 297–306 (2006).

[33] Masani, K., Vette, A. H. & Popovic, M. R. Controlling balance during quiet standing: proportional and derivative controller generates preceding motor command to body sway position observed in experiments. *Gait Post.* **23**, 164–172 (2006). 10.1016/j.gaitpost.2005.01.006 16399512

[34] McIntyre, J. & Bizzi, E. Servo hypotheses for the biological control of movement. *J. Motor Behav.* **25**, 193–202 (1993). 10.1080/00222895.1993.9942049 12581989

[35] Morasso, P., Cherif, A. & Zenzeri, J. Quiet standing: The single inverted pendulum model is not so bad after all. *PloS one* **14**, e0213870 (2019). 30897124 10.1371/journal.pone.0213870 PMC6428281

[36] Bilgin, N. & Kemal Özgören, M. Estimation of the balance-keeping control law applied by a human being upon a sudden sagittal tilt perturbation. *J. Biomech. Eng.* **141** (2019). 10.1115/1.4042683 30758510

[37] van der Kooij, H., Jacobs, R., Koopman, B. & van der Helm, F. An adaptive model of sensory integration in a dynamic environment applied to human stance control. *Biol. Cybern.* **84**, 103–115 (2001). 11205347 10.1007/s004220000196

[38] Todorov, E. Optimality principles in sensorimotor control. *Nat. Neurosci.* **7**, 907–915 (2004). 15332089 10.1038/nn1309 PMC1488877

[39] Davoodi, A., Mohseni, O., Seyfarth, A. & Sharbafi, M. A. From template to anchors: Transfer of virtual pendulum posture control balance template to adaptive neuromuscular gait model increases walking stability. *R. Soc. Open Sci.* **6**, 181911 (2019). 31032044 10.1098/rsos.181911 PMC6458364

[40] Park, S., Horak, F. B. & Kuo, A. D. Postural feedback responses scale with biomechanical constraints in human standing. *Exp. Brain Res.* **154**, 417–427 (2004). 14618285 10.1007/s00221-003-1674-3

[41] Jiang, P., Chiba, R., Takakusaki, K. & Ota, J. A postural control model incorporating multisensory inputs for maintaining a musculoskeletal model in a stance posture. *Adv. Robot.* **31**, 55–67 (2017).

[42] Shanbhag, J. et al. A sensorimotor enhanced neuromusculoskeletal model for simulating postural control of upright standing. *Front. Neurosci.* **18**, 1393749 (2024). 38812972 10.3389/fnins.2024.1393749 PMC11133552

[43] Koelewijn, A. D. & Ijspeert, A. J. Exploring the contribution of proprioceptive reflexes to balance control in perturbed standing. *Front. Bioeng. Biotechnol.* **8**, 866 (2020). 32984265 10.3389/fbioe.2020.00866 PMC7485384

[44] Afschrift, M., Jonkers, I., De Schutter, J. & De Groote, F. Mechanical effort predicts the selection of ankle over hip strategies in nonstepping postural responses. *J. Neurophysiol.* **116**, 1937–1945 (2016). 27489362 10.1152/jn.00127.2016 PMC5144705

[45] Kaminishi, K., Chiba, R., Takakusaki, K. & Ota, J. Investigation of the effect of tonus on the change in postural control strategy using musculoskeletal simulation. *Gait Posture* **76**, 298–304 (2020). 31884257 10.1016/j.gaitpost.2019.12.015

[46] Darici, O. & Kuo, A. D. Humans plan for the near future to walk economically on uneven terrain. *Proc. Natl. Acad. Sci.* **120**, e2211405120 (2023). 37126717 10.1073/pnas.2211405120 PMC10175744

[47] Aftab, Z., Robert, T. & Wieber, P.-B. Balance recovery prediction with multiple strategies for standing humans. *PloS one* **11**, e0151166 (2016). 26974820 10.1371/journal.pone.0151166 PMC4790971

[48] Blakemore, S.-J., Frith, C. D. & Wolpert, D. M. The cerebellum is involved in predicting the sensory consequences of action. *Neuroreport* **12**, 1879–1884 (2001). 11435916 10.1097/00001756-200107030-00023

[49] Ito, M. Bases and implications of learning in the cerebellum adaptive control and internal model mechanism. *Prog. Brain Res.* **148**, 95–109 (2005). 15661184 10.1016/S0079-6123(04)48009-1

[50] Kawato, M. & Wolpert, D. Internal models for motor control. In *Novartis Foundation Symposium 218-Sensory Guidance of Movement: Sensory Guidance of Movement: Novartis Foundation Symposium *. Vol. 218. 291–307 (Wiley Online Library, 2007). 10.1002/9780470515563.ch16 9949827

[51] Kawato, M., Ohmae, S., Hoang, H. & Sanger, T. 50 years since the Marr, Ito, and Albus models of the cerebellum. *Neuroscience* **462**, 151–174 (2021). 32599123 10.1016/j.neuroscience.2020.06.019

[52] Gomi, H. & Kawato, M. Equilibrium-point control hypothesis examined by measured arm stiffness during multijoint movement. *Science* **272**, 117–120 (1996). 8600521 10.1126/science.272.5258.117

[53] Aftab, Z., Robert, T. & Wieber, P.-B. Predicting multiple step placements for human balance recovery tasks. *J. Biomech.* **45**, 2804–2809 (2012). 22999377 10.1016/j.jbiomech.2012.08.038

[54] Shen, K., Chemori, A. & Hayashibe, M. Effectiveness evaluation of arm usage for human quiet standing balance recovery through nonlinear model predictive control. In *2020 3rd International Conference on Control and Robots (ICCR)*. 150–153 (IEEE, 2020).

[55] Inkol, K. A. & McPhee, J. Simulating human upper and lower limb balance recovery responses using nonlinear model predictive control. In *2021 43rd Annual International Conference of the IEEE Engineering in Medicine & Biology Society (EMBC)*. 4717–4721 (IEEE, 2021). 10.1109/EMBC46164.2021.9630208 34892265

[56] Castano, J. A., Zhou, C., Li, Z. & Tsagarakis, N. Robust model predictive control for humanoids standing balancing. In *2016 International Conference on Advanced Robotics and Mechatronics (ICARM)*. 147–152 (IEEE, 2016).

[57] Shen, K., Chemori, A. & Hayashibe, M. Reproducing human arm strategy and its contribution to balance recovery through model predictive control. *Front. Neurorobot.* **15**, 679570 (2021). 34079448 10.3389/fnbot.2021.679570 PMC8165250

[58] Taga, G., Yamaguchi, Y. & Shimizu, H. Self-organized control of bipedal locomotion by neural oscillators in unpredictable environment. *Biol. Cybern.* **65**, 147–159 (1991). 1912008 10.1007/BF00198086

[59] Li, Y. & Levine, W. S. An optimal model predictive control model for human postural regulation. In *2009 17th Mediterranean Conference on Control and Automation*. 1143–1148 (IEEE, 2009).

[60] Grasso, C., Orsini, P., Bruschini, L., Manzoni, D. & Barresi, M. A new technique to investigate vestibulospinal reflexes. *Arch. Ital. Biol.* **151**, 54–66 (2013). 24442983 10.4449/aib.v151i2.1487

[61] Jeka, J., Kiemel, T., Binder, M., Hirokawa, N. & Windhorst, U. Modeling of human postural control. In *Encyclopedia of Neuroscience.* 2381–2384 (Springer, 2009).

[62] Kim, S., Horak, F. B., Carlson-Kuhta, P. & Park, S. Postural feedback scaling deficits in Parkinson’s disease. *J. Neurophysiol.* **102**, 2910–2920 (2009). 19741108 10.1152/jn.00206.2009 PMC2777824

[63] Shen, K., Chemori, A. & Hayashibe, M. Human-like balance recovery based on numerical model predictive control strategy. *IEEE Access* **8**, 92050–92060 (2020).

[64] Darby, A., Brownjohn, J., Shahabpoor, E. & Heshmati, K. Vsimulators: A new UK-based immersive experimental facility for studying occupant response to wind-induced motion of tall buildings. *Int. J. High-Rise Build.* **11**, 347–362 (2022).

[65] Blenkinsop, G. M., Pain, M. T. & Hiley, M. J. Balance control strategies during perturbed and unperturbed balance in standing and handstand. *R. Soc. Open Sci.* **4**, 161018 (2017). 28791131 10.1098/rsos.161018 PMC5541526

[66] Ballreich, C. VRML Humanoid Model (1997).

[67] Tordoff, B. & Mayol, W. MATLAB Humanoid Model. In Technical Report, Robotics Research Group, University of Oxford (2002). https://www.robots.ox.ac.uk/wmayol/3D/nancy_matlab.html.

[68] Kudoh, S., Komura, T. & Ikeuchi, K. The dynamic postural adjustment with the quadratic programming method. In *IEEE/RSJ International Conference on Intelligent Robots and Systems*. Vol. 3. 2563–2568 (IEEE, 2002).

[69] Stephens, B. Integral control of humanoid balance. In *2007 IEEE/RSJ International Conference on Intelligent Robots and Systems*. 4020–4027 (IEEE, 2007).

[70] Guihard, M. & Gorce, P. Dynamic control of bipeds using ankle and hip strategies. In *IEEE/RSJ International Conference on Intelligent Robots and Systems*. Vol. 3. 2587–2592 (IEEE, 2002).

[71] Atkeson, C. G. & Stephens, B. Multiple balance strategies from one optimization criterion. In *2007 7th IEEE-RAS International Conference on Humanoid Robots*. 57–64 (IEEE, 2007).

[72] Suzuki, Y., Nomura, T., Casadio, M. & Morasso, P. Intermittent control with ankle, hip, and mixed strategies during quiet standing: A theoretical proposal based on a double inverted pendulum model. *J. Theor. Biol.* **310**, 55–79 (2012). 22732276 10.1016/j.jtbi.2012.06.019

[73] Aftab, Z., Robert, T. & Wieber, P.-B. Ankle, hip and stepping strategies for humanoid balance recovery with a single model predictive control scheme. In *2012 12th IEEE-RAS International Conference on Humanoid Robots (Humanoids 2012)*. 159–164 (IEEE, 2012).

[74] Jones, R., Ratnakumar, N., Akbaş, K. & Zhou, X. Delayed center of mass feedback in elderly humans leads to greater muscle co-contraction and altered balance strategy under perturbed balance: A predictive musculoskeletal simulation study. *Plos one* **19**, e0296548 (2024). 38787871 10.1371/journal.pone.0296548 PMC11125460

[75] Wan, G., Wang, P., Han, Y. & Liang, J. Torque modulation mechanism of the knee joint during balance recovery. *Comput. Biol. Med.* **175**, 108492 (2024). 38678940 10.1016/j.compbiomed.2024.108492

[76] Farris, D. J., Birch, J. & Kelly, L. Foot stiffening during the push-off phase of human walking is linked to active muscle contraction, and not the windlass mechanism. *J. R. Soc. Interface* **17**, 20200208 (2020). 32674708 10.1098/rsif.2020.0208 PMC7423437

[77] Birch, J. V., Kelly, L. A., Cresswell, A. G., Dixon, S. J. & Farris, D. J. Neuromechanical adaptations of foot function to changes in surface stiffness during hopping. *J. Appl. Physiol.* **130**, 1196–1204 (2021). 33571058 10.1152/japplphysiol.00401.2020

[78] Marelli, S. et al. The effects of altering the center of pressure in standing subjects exposed to foot-transmitted vibration on an optimized lumped-parameter model of the foot. *Vibration* **4**, 893–905 (2021).

[79] Goggins, K. A. et al. Four degree-of-freedom lumped parameter model of the foot-ankle system exposed to vertical vibration from 10 to 60 hz with varying centre of pressure conditions. *Ergonomics* **64**, 1002–1017 (2021). 33688787 10.1080/00140139.2021.1891298

[80] Chadefaux, D. et al. Development of a two-dimensional dynamic model of the foot-ankle system exposed to vibration. *J. Biomech.* **99**, 109547 (2020). 31831138 10.1016/j.jbiomech.2019.109547

[81] Latash, M. L. & Zatsiorsky, V. M. Joint stiffness: Myth or reality?. *Hum. Mov. Sci.* **12**, 653–692 (1993).

[82] Rouse, E. J., Gregg, R. D., Hargrove, L. J. & Sensinger, J. W. The difference between stiffness and quasi-stiffness in the context of biomechanical modeling. *IEEE Trans. Biomed. Eng.* **60**, 562–568 (2012). 23212310 10.1109/TBME.2012.2230261 PMC4266141

[83] Cerda-Lugo, A., González, A., Cardenas, A. & Piovesan, D. Modeling the neuro-mechanics of human balance when recovering from a fall: A continuous-time approach. *Biomed. Eng. Online* **19**, 1–24 (2020). 32867771 10.1186/s12938-020-00811-1 PMC7457816

