# Supplementary material for: Humans self-organise balance control strategies on a dynamic platform
Source: Sci Rep. 2025 Jul 8;15:24366. doi: 10.1038/s41598-025-09127-3 (PMC12238653; doi:10.1038/s41598-025-09127-3)
Supplement: Supplementary file 2 — Supplementary Information. [file 41598_2025_9127_MOESM2_ESM.pdf]

Supplementary Information: Humans Self-Organise Balance Control Strategies on A Dynamic Platform

Naser Taleshi[1,*], Amid Kheirandish[1], James MW Brownjohn[2], Sarah E Lamb[3], and Genevieve KR Williams[1]

[1]Department of Public Health and Sport Sciences, Faculty of Health and Life Sciences, University of Exeter, Exeter, UK
[2]Department of Engineering, Faculty of Environment, Science and Economy, University of Exeter, Exeter, UK
[3]University of Exeter Medical School, Faculty of Health and Life Sciences, University of Exeter, Exeter, UK
[*]n.taleshi@exeter.ac.uk

Plant:body dynamics model

Equation of motion

The body dynamics can be derived in matrix form as Eq. S1 using the Newton-Euler method [7]. Segment parameters, Table S1, are also considered based on average male data [2,4].

$$
\begin{aligned}
\overbrace{\begin{bmatrix} 1 & -1 & 0 \\ 0 & 1 & -1 \\ 0 & 0 & 1 \end{bmatrix}}^{E(\theta)} \begin{bmatrix} \tau_1 \\ \tau_2 \\ \tau_3 \end{bmatrix} = & \overbrace{\begin{bmatrix} d_{11} & d_{12}\cos(\theta_1-\theta_2) & d_{13}\cos(\theta_1-\theta_3) \\ d_{12}\cos(\theta_1-\theta_2) & d_{22} & d_{23}\cos(\theta_2-\theta_3) \\ d_{13}\cos(\theta_1-\theta_3) & d_{23}\cos(\theta_2-\theta_3) & d_{33} \end{bmatrix}}^{DD(\theta)} \begin{bmatrix} \ddot{\theta}_1 \\ \ddot{\theta}_2 \\ \ddot{\theta}_3 \end{bmatrix} \\
& + \overbrace{\begin{bmatrix} 0 & d_{12}\,\sin(\theta_1-\theta_2) & d_{13}\sin(\theta_1-\theta_3) \\ -d_{12}\,\sin(\theta_1-\theta_2) & 0 & +d_{23}\sin(\theta_2-\theta_3) \\ -d_{13}\sin(\theta_1-\theta_3) & -d_{23}\sin(\theta_2-\theta_3) & 0 \end{bmatrix}}^{H(\theta,\dot{\theta})} \begin{bmatrix} \dot{\theta}_1^2 \\ \dot{\theta}_2^2 \\ \dot{\theta}_3^2 \end{bmatrix} \\
& - \overbrace{\begin{bmatrix} f_1 g & 0 & 0 \\ 0 & f_2 g & 0 \\ 0 & 0 & f_3 g \end{bmatrix}}^{G(\theta)} \begin{bmatrix} \cos(\theta_1) \\ \cos(\theta_2) \\ \cos(\theta_3) \end{bmatrix}
\end{aligned} \tag{S1}
$$

Let $\theta = \begin{bmatrix}\theta_1 \; \theta_2 \; \theta_3\end{bmatrix}^T$ represent the ankle, knee, and hip joint angles (from horizontal), and let $\dot{\theta} = \begin{bmatrix}\dot{\theta}_1 \; \dot{\theta}_2 \; \dot{\theta}_3\end{bmatrix}^T$ represent their respective angular velocities. The model's dynamics can be compactly expressed as follows (see Eq. S2).

Table S1: Parameters of the Model

Parameter	m (kg)	l (m)	r (m)	I (kgm^2)	Derived Parameters
Shank (m_1, l_1, r_1, I_1)	6.14	0.43	0.25	0.11	$d_{11} = m_1 r_1^2 + (m_2+m_3) l_1^2 + I_1$<br>$d_{22} = m_2 r_2^2 + m_3 l_2^2 + I_2$
Thigh (m_2, l_2, r_2, I_2)	13.2	0.43	0.25	0.26	$d_{12} = f_2 l_1$, $d_{23} = l_2 f_3$
HAT (m_3, l_3, r_3, I_3)	44.75	0.83	0.31	7.53	$d_{13} = l_1 f_3$, $d_{33} = m_3 r_3^2 + I_3$
Foot (m_f, l_f)	1.91	0.27	–	–	–
Foot Measurements (a, b, c)	–	–	0.05, 0.07, 0.08	–	–

$$E(\theta)\tau = DD(\theta)\ddot{\theta} + H(\theta,\dot{\theta})\dot{\theta} + G(\theta) \tag{S2}$$

$DD(\theta)\ddot{\theta}$ denotes inertial torques from body inertia. $H(\theta,\dot{\theta})\dot{\theta}$ and $G(\theta)$ represent nonlinear Coriolis, and gravitational torques, respectively. The matrix $\tau = \begin{bmatrix}\tau_1 \; \tau_2 \; \tau_3\end{bmatrix}^T$ and $E(\theta)$ denote input torques applied at joints and a distribution matrix, respectively. To investigate the relative motion of the COP and COM, as well as to impose biomechanical constraints on our controller, it is necessary to calculate the COP, COM, and ground reaction force (GRF). The GRF in horizontal and vertical direction are obtained as:

$$\begin{aligned} F_{gx} = & -f_1\left(\ddot{\theta}_1 \sin\theta_1 \;+ \dot{\theta}_1^2 \cos\theta_1\right) - f_2\left(\ddot{\theta}_2 \sin\theta_2 + \dot{\theta}_2^2 \cos\theta_2\right) \\ & \quad - f_3\left(\ddot{\theta}_3 \sin\theta_3 + \dot{\theta}_3^2 \cos\theta_3\right) - m_f a_f \end{aligned} \tag{S3}$$

$$\begin{aligned} F_{gy} = & f_1\;(\ddot{\theta}_1 \cos\theta_1 - \dot{\theta}_1^2 \sin\theta_1) + f_2\,(\ddot{\theta}_2 \cos\theta_2 - \dot{\theta}_2^2 \sin\theta_2) \\ & + f_3\left[\ddot{\theta}_3 \cos\theta_3 - \dot{\theta}_3^2 \sin\theta_3\right] + (m_3 + m_2 + m_1 + m_f)\,g \end{aligned} \tag{S4}$$

COM position in horizontal and vertical direction can be obtained via:

$$COM_x = \frac{f_1\cos(\theta_1) + f_2\cos(\theta_2) + f_3\cos(\theta_3) - m_f c}{M} \tag{S5}$$

$$COM_y = \frac{f_1\sin(\theta_1) + f_2\sin(\theta_2) + f_3\sin(\theta_3) - m_f(\frac{b}{2})}{M} \tag{S6}$$

and finally, COP in the AP direction can be expressed as:

$$COP_x = \frac{-\tau_1 + c m_f g + bF_{gx} - (l_f - a)F_{gy}}{F_{gy}} \tag{S7}$$

Controller

Design of MPC frameworks rely on a mathematical model of the plant, often in state-space form [5, 8]. It includes state variables evolving over time via linear differential equations, with input and output variables linked through linear equations. The state variables (x) fully describe the system's dynamics, allowing predictions of its future behaviour via current state information. Therefore, to design MPC, the nonlinear system described in Eq. S1 needs linearization and conversion into state-space form. During standing balance, joint angles undergo small changes, typically within 15 degrees or 0.26 radians, allowing for the small-angle approximation[3]. Thus, with $\varphi(t) = \theta(t) - \pi/2$, trigonometric ratios can be simplified: $\cos\varphi \approx 1$, $\tan\varphi$, $\sin\varphi$, and $\varphi \approx 0$. This simplifies Eq. S1 as:

$$\overbrace{\begin{bmatrix} 1 & -1 & 0 \\ 0 & 1 & -1 \\ 0 & 0 & 1 \end{bmatrix}}^{E(\varphi)} \begin{bmatrix} \tau_1 \\ \tau_2 \\ \tau_3 \end{bmatrix} = \overbrace{\begin{bmatrix} d_{11} & d_{12} & d_{13} \\ d_{12} & d_{22} & d_{23} \\ d_{13} & d_{23} & d_{33} \end{bmatrix}}^{D(\varphi)} \begin{bmatrix} \ddot{\varphi}_1 \\ \ddot{\varphi}_2 \\ \ddot{\varphi}_3 \end{bmatrix} + \overbrace{\begin{bmatrix} f_1 g & 0 & 0 \\ 0 & f_2 g & 0 \\ 0 & 0 & f_3 g \end{bmatrix}}^{G(\varphi)} \begin{bmatrix} \sin(\varphi_1) \\ \sin(\varphi_2) \\ \sin(\varphi_3) \end{bmatrix} \tag{S8}$$

and rearranging Eq. S8 converts it into following form:

$$\frac{d}{dt}\begin{bmatrix} \varphi \\ \dot{\varphi} \end{bmatrix} = \overbrace{\begin{bmatrix} 0 & I \\ -D^{-1}(\varphi)\frac{dG(\varphi)}{d\varphi} & 0 \end{bmatrix}}^{A_m} \begin{bmatrix} \varphi \\ \dot{\varphi} \end{bmatrix} + \overbrace{\begin{bmatrix} 0 \\ D^{-1}(\varphi)E(\varphi) \end{bmatrix}}^{B_m} \tau \tag{S9}$$

As shown in Eq. S10, the body dynamics are fully characterized by joint angular motion (φ) and velocity ($\dot{\varphi}$), where $\varphi = [\varphi_1\ \varphi_2\ \varphi_3]^T$ and $\dot{\varphi} = [\dot{\varphi}_1\ \dot{\varphi}_2\ \dot{\varphi}_3]^T$. Thus, ankle, knee, and hip joint motion and velocity serve as state variables and sensory feedback to the CNS. Defining the state vector as $x(t) = [\varphi\ \dot{\varphi}]^T$, Eq. S9 can be linearized and expressed in the standard state-space form required for the controller as follows:

$$\dot{x}(t) = A_m x(t) + B_m \tau(t) \tag{S10}$$

the output equation is also given as:

$$y(t) = C_m x(t) \tag{S11}$$

The control variable τ represents joint torques for the ankle, knee, and hip joints. The state variable vector $x(t)$ includes six states for joint angular motion and velocity. The process output is determined by y . A_m, B_m, and C_m matrices, describe system underlying dynamics, control variables effect on the system states, and state-output relationship. In the absence of state noise, the states (x and $\dot{x}$) directly influence the process output y. Thus, without noise, the output equals the states, i.e., $y(t) = x(t)$, or C_m is the identity matrix.

MPC algorithm

We employed a discrete-time MPC formulation with boundary conditions for our balance recovery problem [12]. The state vector x_m and the control input vector τ are defined as follows:

$$\tau = \begin{bmatrix} \tau_1 \\ \tau_2 \\ \tau_3 \end{bmatrix} = \begin{bmatrix} \tau_a \\ \tau_k \\ \tau_h \end{bmatrix}, \quad x_m = \begin{bmatrix} x_1 \\ x_2 \\ x_3 \\ x_4 \\ x_5 \\ x_6 \end{bmatrix} = \begin{bmatrix} \varphi_1 \\ \varphi_2 \\ \varphi_3 \\ \dot{\varphi}_1 \\ \dot{\varphi}_2 \\ \dot{\varphi}_3 \end{bmatrix} = \begin{bmatrix} \varphi_a \\ \varphi_k \\ \varphi_h \\ \dot{\varphi}_a \\ \dot{\varphi}_k \\ \dot{\varphi}_h \end{bmatrix} \tag{S12}$$

The discrete-time state-space model, which is derived from the continuous-time state-space model presented in S10 and S11, is given by:

$$x_m(k+1) = A_m x_m(k) + B_m \tau(k) \tag{S13}$$

$$y(k) = C_m x_m(k) \tag{S14}$$

Our plant model takes $\tau(k)$ as its input. To meet control design requirements following the receding horizon control principle of MPC, the model must incorporate an integrator, resulting in an augmented state-space model.

0.1.1 Augmented state-space model

To express the discrete-time state-space model given by Eq. S13 and Eq. S14 in differential form, we introduce a new state variable defined as $x(k) = [\ \Delta x_m(k)^T \quad y(k)\ ]^T$. The discrete-time state-space model, expressed in differential form, can be then represented as:

$$\begin{aligned} x(k+1) &= Ax(k) + B\Delta\tau(k) \\ y(k) &= Cx(k) \end{aligned} \tag{S15}$$

where

$$x(k+1) = \begin{bmatrix} \Delta x_m(k+1) \\ y(k+1) \end{bmatrix}, \quad x(k) = \begin{bmatrix} \Delta x_m(k) \\ y(k) \end{bmatrix}, \quad A = \begin{bmatrix} A_m & O_m^T \\ C_m A_m & I_{q\times q} \end{bmatrix}, \quad B = \begin{bmatrix} B_m \\ C_m B_m \end{bmatrix}, \quad C = \begin{bmatrix} O_m & I_{q\times q} \end{bmatrix}. \tag{S16}$$

This augmented form model (A, B, C) is essential for predictive control, facilitating system dynamics description and control strategy formulation. Matrices have the following dimensions: $I_{q\times q}$ is a 6×6 identity matrix (where q is outputs). O_m is a 6×6 and 3×1 zero matrix. A_m, B_m, and C_m are $n_1 \times n_1$ (6×6), $n_1 \times m$ (6×3), and $q\times n_1$ (6×6), respectively (where n_1 is states, and m is control signals).

0.1.2 Prediction of future state and output variables (joint recovery motion)

After formulating the mathematical model, the next step is predicting plant output (joint motions) using future control variables (torques) within an optimization window, known as receding horizon predictive control. The MPC problem is an iterative optimal control problem with a finite horizon. At sampling instant k_i ($k_i > 0$), the state variable vector $x(k_i)$ provides current plant information. Using the augmented state-space model (A, B, C), future state variables can be predicted sequentially for N_p samples as follows:

$$\begin{aligned}
x(k_i+1|k_i) =& Ax(k_i) + B\Delta\tau(k_i) \\
x(k_i+2|k_i) =& Ax(k_i+1|k_i) + B\Delta\tau(k_i+1) \\
=& A^2x(k_i) + AB\Delta\tau(k_i) + B\Delta\tau(k_i+1) \\
& \vdots \\
x(k_i+N_p|k_i) =& A^{N_p}x(k_i) + A^{N_p-1}B\Delta\tau(k_i) + A^{N_p-2}B\Delta\tau(k_i+1) \\
& + \ldots + A^{N_p-N_c}B\Delta\tau(k_i+N_c-1)
\end{aligned} \tag{S17}$$

$x(k_i+m|k_i)$ refers to the predicted state variable at k_i+m, based on current plant info $x(k_i)$. "Prediction horizon" N_p represents the duration for which the controller predicts the future dynamics, while control horizon N_c determines the duration during which the controller applies control actions to the system. The prediction and control horizon depend on application and system properties, with N_c usually less than or equal to N_p. As mentioned, without noise, predicted output variables (process output) are derived from predicted state variables, i.e $y(k) = x(k)$. The Eq. S17 can be expressed in a compact matrix form as:

$$Y = Fx(k_i) + \Phi\Delta\Gamma \tag{S18}$$

where

$$F = \begin{bmatrix} A \\ A^2 \\ \vdots \\ A^{N_p} \end{bmatrix}; \Phi = \begin{bmatrix} B & 0 & 0 & \cdots & 0 \\ AB & B & 0 & \cdots & 0 \\ A^2B & AB & B & \cdots & 0 \\ A^{N_p-1}B & A^{N_p-2}B & A^{N_p-3}B & \cdots & A^{N_p-N_c}B \end{bmatrix}; \tag{S19}$$

$$\Gamma = [\Delta\tau(k_i) \;\; \Delta\tau(k_i+1) \;\; \Delta\tau(k_i+2)...\Delta\tau(k_i+N_c-1)]^T$$

Predicted variables in Eq. S17 depend on current state $x(k_i)$ and future control actions $\Delta\tau(k_i+j)$. Assuming availability of current plant information $x(k_i)$ via measurement, predicting future state y and output x (φ and $\dot{\varphi}$) requires only determining future control actions $\Delta\tau(k_i+j)$. The MPC algorithm achieves this using optimization techniques, allowing proactive assessment of movement trajectory and adherence to operational constraints.

0.1.3 Constraints on human motor control system

During optimization process, we consider balance system constraints which are common in optimisation-based postural control models including MPC framework, as previously noted in [1, 9–11].

1. The first constraint addresses control command limits $\tau(k)$, considering the human body's torque production limits. For $i = 1, 2, 3$, torques should meet

$$\tau_{min}(i) \leq \tau(k_i) \leq \tau_{max}(i) \tag{S20}$$

where $\tau_{min}(1) = 20\ [Nm]$, $\tau_{min}(2) = 40\ [Nm]$, $\tau_{min}(3) = 100\ [Nm]$ are the minimum ankle, knee and hip joint torques, respectively, we can produce in backward direction, while $\tau_{max}(1) = 20\ [Nm]$, $\tau_{max}(2) = 40\ [Nm]$, $\tau_{max}(3) = 100[Nm]$ are the max ankle, knee and hip joint torques, respectively, we can produce in forward direction [10].

2. The second type of constraint deals with output $y(k)$ or state variable $x(k)$ constraints as ranges of human joint movement is limited due to cartilages and ligaments. For all $i = 1, 2, .., 6$, the system states should satisfy:

$$x_{min}(i) \leq x(k_i) \leq x_{max}(i) \tag{S21}$$

where $x_{min}(1) = 40\ [Deg]$, $x_{min}(2) = -30\ [Deg]$, $x_{min}(3) = 90\ [Deg]$ are the minimum, while $x_{max}(1) = 110\ [Deg]$, $x_{max}(2) = 90\ [Deg]$, $x_{max}(3) = 150\ [Deg]$ are the maximum angular motion of ankle, knee and hip joint, respectively [10]. The boundaries for angular velocity of the joints, $x_{min}(i), x_{max}(i)$ in $[Deg/s]$ for $i = 4, 5, 6$ are defined based on our experimental results.

3. As we aimed to investigate balance control strategies without stepping, the COM and COP must reside within the length of the BOS.

$$\begin{aligned} -a\ &\leq COP(k_i) \leq l_f - a \\ -a\ &\leq COM(k_i) \leq l_f - a \end{aligned} \tag{S22}$$

4. Here, two more constraints on gravitational force are considered in MPC design to get more human-like recovery motion, [2, 6].

   - Friction constraint: our participants did not slipped on the platform, so the horizontal GRF F_{gx} must not exceed the slip threshold dictated by the coefficient of friction, μ. In this study, coefficient of friction $\mu = 0.6$ is considered to simulate frictional properties of our support surface.

   $$F_{gx}(k_i) < \mu F_{gy}(k_i) \tag{S23}$$

   - The second constraint is the gravity constraint which ensures that the net vertical ground reaction force is positive.

   $$0 \leq F_{gy}(k_i) \tag{S24}$$

We used a closed-loop MPC model that compares desired posture (upright stance) with sensory feedback information of actual movement. Through optimization in short time windows, the model corrects errors between the desired posture and actual movements. The signal $r(k)$, representing zero joint motion or velocity, serves as the desired posture (upright stance). Assuming $r(k_i)$, the desired posture, is constant during prediction horizon (N_p) at k_i, we define R_s, a data vector with this information, as follows:

$$R_s = \overbrace{\begin{bmatrix} 1 & 1 & \cdots & 1 \end{bmatrix}}^{N_p} r(k_i) = \bar{R}_s r(k_i) \tag{S25}$$

The predictive control system's goal is to minimize the error between desired posture $r(k_i)$ and predicted output over (N_p). It finds optimal control $\Delta\Gamma$ to minimize the cost function between set-point and output, aligning joints with the upright stance.

Supplementary movie

Movie S1

Movie S1 is an animation that shows the interaction of a human model with a sinusoidally moving platform as its frequency increases. Red and blue markers represent the COM and COP, respectively, while the red line indicates the desired upright postural position. The simulation demonstrates the transition from ankle to knee strategy in response to increasing platform frequency.

References

[1] Zohaib Aftab, Thomas Robert, and Pierre-Brice Wieber. Balance recovery prediction with multiple strategies for standing humans. *PloS one*, 11(3):e0151166, 2016.

[2] Kamran Iqbal and Yi-Chung Pai. Predicted region of stability for balance recovery:: motion at the knee joint can improve termination of forward movement. *Journal of biomechanics*, 33(12):1619–1627, 2000.

[3] Gon Khang and Fefix E Zajac. Paraplegic standing controlled by functional neuromuscular stimulation. ii. computer simulation studies. *IEEE transactions on biomedical engineering*, 36(9):885–894, 1989.

[4] Arthur D Kuo and Felix E Zajac. A biomechanical analysis of muscle strength as a limiting factor in standing posture. *Journal of Biomechanics*, 26:137–150, 1993.

[5] Jay H Lee. Model predictive control: Review of the three decades of development. *International Journal of Control, Automation and Systems*, 9:415–424, 2011.

[6] Yi-Chung Pai and James Patton. Center of mass velocity-position predictions for balance control. *Journal of biomechanics*, 30(4):347–354, 1997.

[7] D Gordon E Robertson, Graham E Caldwell, Joseph Hamill, Gary Kamen, and Saunders Whittlesey. *Research methods in biomechanics*. Human kinetics, 2013.

[8] Max Schwenzer, Muzaffer Ay, Thomas Bergs, and Dirk Abel. Review on model predictive control: An engineering perspective. *The International Journal of Advanced Manufacturing Technology*, 117(5-6):1327–1349, 2021.

[9] Keli Shen, Ahmed Chemori, and Mitsuhiro Hayashibe. Effectiveness evaluation of arm usage for human quiet standing balance recovery through nonlinear model predictive control. In *2020 3rd International Conference on Control and Robots (ICCR)*, pages 150–153. IEEE, 2020.

[10] Keli Shen, Ahmed Chemori, and Mitsuhiro Hayashibe. Human-like balance recovery based on numerical model predictive control strategy. *IEEE Access*, 8:92050–92060, 2020.

[11] Keli Shen, Ahmed Chemori, and Mitsuhiro Hayashibe. Reproducing human arm strategy and its contribution to balance recovery through model predictive control. *Frontiers in neurorobotics*, 15:679570, 2021.

[12] Liuping Wang. *Model predictive control system design and implementation using MATLAB®*. Springer Science & Business Media, 2009.
